# Creating a replicable, valid cross-platform buffering technique: The sausage network buffer for measuring food and physical activity built environments

**DOI:** 10.1186/1476-072X-11-14

**Published:** 2012-07-09

**Authors:** Ann Forsyth, David Van Riper, Nicole Larson, Melanie Wall, Dianne Neumark-Sztainer

**Affiliations:** 1 Department of City and Regional Planning, Cornell University, Ithaca, NY, USA; 2 Minnesota Population Center, University of Minnesota, Minneapolis, MN, USA; 3 Division of Epidemiology and Community Health, School of Public Health, University of Minnesota, Minneapolis, MN, USA; 4 Division of Biostatistics, Department of Psychiatry and Department of Biostatistics, Mailman School of Public Health, Columbia University, New York, NY, USA

**Keywords:** Buffer, Network, Obesity, Physical activity environments, Food environments, GIS, Reliability, Validity, Utility

## Abstract

**Background:**

Obesity researchers increasingly use geographic information systems to measure exposure and access in neighborhood food and physical activity environments. This paper proposes a network buffering approach, the “sausage” buffer. This method can be consistently and easily replicated across software versions and platforms, avoiding problems with proprietary systems that use different approaches in creating such buffers.

**Methods:**

In this paper, we describe how the sausage buffering approach was developed to be repeatable across platforms and places. We also examine how the sausage buffer compares with existing alternatives in terms of buffer size and shape, measurements of the food and physical activity environments, and associations between environmental features and health-related behaviors. We test the proposed buffering approach using data from EAT 2010 (Eating and Activity in Teens), a study examining multi-level factors associated with eating, physical activity, and weight status in adolescents (n = 2,724) in the Minneapolis/St. Paul metropolitan area of Minnesota.

**Results:**

Results show that the sausage buffer is comparable in area to the classic ArcView 3.3 network buffer particularly for larger buffer sizes. It obtains similar results to other buffering techniques when measuring variables associated with the food and physical activity environments and when measuring the correlations between such variables and outcomes such as physical activity and food purchases.

**Conclusions:**

Findings from various tests in the current study show that researchers can obtain results using sausage buffers that are similar to results they would obtain by using other buffering techniques. However, unlike proprietary buffering techniques, the sausage buffer approach can be replicated across software programs and versions, allowing more independence of research from specific software.

## Background

Accurately capturing exposure and access to different food and physical activity environments is of great interest to researchers engaged in identifying environmental correlates of dietary patterns, physical activity and obesity. In the past two decades GIS techniques have been increasingly used for these purposes [1]. In order to understand environmental contexts of populations, researchers have often used “buffers” of a certain distance around key environments such as homes, schools, work sites, and parks [2].

Straight line buffers (also called circular, crow flies, or airline buffers) simply go out a certain distance in a straight line from the facility or place, creating a circle (if the item being buffered is a point). In contrast, network or street distance buffers try to more closely approximate the experience of moving around an area by measuring out a certain distance on the street network and then using some method of joining the dots. Network buffers have been made easy to use in the past decade and a half by algorithms in GIS programs; previous to the development of these algorithms, creating them manually was a tedious task. However, in the mid-2000s, Esri ArcGIS software abruptly changed its method for calculating such buffers making older analyses not directly comparable to recent assessments. Esri (originally Environmental Systems Research Institute) is the dominant developer of GIS software with a 30 % market share internationally in 2009, almost double that of its closest competitor, Integraph [3]. This change, made by a popular GIS developer, highlights the proprietary nature of much GIS software and its design for professional rather than research contexts.

This paper explores an alternative to using proprietary algorithms—constructing a network buffer from scratch. It answers two questions. First, is it possible to create an approach that can be consistently and easily replicated across software versions and platforms (i.e. hardware and operating systems)? Second, does such an approach accurately measure where people can get to from a starting point and the environments they experience along the way? That is, does it measure relevant parts of the local environment?

In this paper, we describe how the sausage buffering approach was developed to be repeatable across platforms and places. The proposed sausage-shaped buffer, buffers all roads by a consistent dimension (the “radius”) out from the center line. We examine how this approach compares with existing alternatives in terms of (a) size and shape of buffers, (b) measurements of the food and physical activity environments, and (c) correlations between environmental features and health-related behaviors among adolescents.

The sausage buffering approach has three main strengths. First, it is theoretically defensible as it directly measures the environment near the streets or paths along which people travel. Second, it has much in common with other proprietary techniques meaning that it provides comparable data to measures already in existence. Third, it is easily reproducible across GIS platforms and program versions, meaning that it provides a stable and reliable measure that can be used in the future and by those using different GIS programs [4]. In the obesity field, one other study has published such an approach, in a preliminary methods paper without results, called the “street buffer” [5]. However, to the best of our knowledge, no previous studies have systematically compared the sausage-shaped buffer approach with other buffer types, particularly in a health context.

The analysis described here highlights three issues with using GIS in health research that frame the current paper: 1) reliability, 2) validity, and 3) utility across places and datasets. In regards to reliability, the more sophisticated, multifunctional, and widely-used GIS programs have been designed primarily for use in professional practice where the ability to replicate work is not as highly valued as it is in health research. Available documentation for many such GIS programs is not clear about their internal algorithms or even about the definition of terms (as will be noted below, ArcGIS uses some non-standard terminology that it does not fully define in its documentation).

Thus, research users of GIS need to figure out how to create reliable methods that can be used across platforms, both commercial and open source [6]. In terms of food and physical activity environments, most discussions of reliability have focused on survey and audit tools where inter-rater reliability, test-retest reliability, and item consistency are most important [4]. Reliability of GIS buffers instead relates to the repeatability of the measure across programs and platforms. Such repeatability is particularly important in international comparative work. For example, while a few software companies dominate the global market, there is regional variation in market share. Repeatability across platforms is a fundamental prerequisite for comparison. It is this issue of repeatability that led us to develop the sausage buffer, i.e., for the purpose of conducting studies of food and physical activity environments and allowing comparison across time and place.

The second issue is validity. Buffers approximate the environments experienced by populations. They are a great improvement over measures using pre-existing geographies, such as census tracts, to define “neighborhoods” or local areas in that they are centered on the participant. Network buffers go one step further than straight line buffers and approximate the extent of the environment that is experienced by people moving along streets. But it is important to create buffers that closely match the geography of the local food or physical activity environment as it is experienced or perceived by study participants. What do people see, smell, and hear as they walk, cycle, or drive along streets? How far from the street centerline does this experienced environment extend? Does the measurement geography (e.g. the buffer) approximate experience in a way that is relevant for health behaviors? Research on how people perceive their neighborhoods reveals that such perceived neighborhoods vary greatly in size and shape [7]. However, the sausage buffer has face validity in terms of assessing local environments relevant for health behaviors. Like other buffer types, sausage buffers can be centered on such important places as homes, schools, or work sites and capture nearby environments that provide settings or contexts for health-related choices (e.g. local access to green space or healthy food options).

Finally, the third issue relates to what Lytle [4], in reviewing survey and audit tools, calls “other related measurement qualities.” Two examples given in the review completed by Lytle are utility of a tool across (a) populations and (b) health concerns. In the case of GIS and buffering, other relevant issues would be utility across (c) places and (d) datasets. It is important to examine whether a measure makes sense in different kinds of physical environments and with different kinds of street and path network data, such as might be available in different parts of a country or across countries. Again, the sausage buffer should perform as well as other buffering methods, and because of its simplicity it may do better than some. That is, a sausage buffer approach appears to have no disadvantages as compared to other methods and it has strengths in repeatability.

To date, little research in the area of food, physical activity, and obesity has looked explicitly at buffering. Only a few papers have reported results for both straight line and network buffers (e.g. [8,9]). Oliver et al. [10] explicitly compared straight line and network buffers in terms of associations with walking for leisure and for errands, finding the network buffer to be better than the circular buffer for this purpose. Burton et al. [5] published a paper on the methods of the HABITAT longitudinal study of change in physical activity in middle-aged adults in Brisbane and included a diagram of three buffer types: circular, network, and street (the last akin to the sausage buffer). They used MapInfo, another major GIS program, to create the buffers. The current paper builds on this previous research by providing a more substantial discussion of buffering in regards to studies of population health.

## Methods

### Buffer development

We compared the variability of built environment measurements (e.g. access to fast-food restaurants, parks, etc.) using different buffering methods. Specifically, we compared areas and shapes of the traditional network buffers in ArcView 3.3, the newer ArcGIS 9.3 detailed and generalized network buffers, and the proposed sausage buffers. The sausage buffer is created by buffering all roads out a certain “street distance” from the starting point, and for a “radius” of some number of meters on each side of the road center line. Details about GIS steps are included in a protocols document available online [11].^1^ In addition to comparing buffer types in terms of size and shape, we also examined different options for sausage buffers including end point shapes, end location, and different trim options for the generalized and detailed buffers.

### Buffer testing

This paper uses data from EAT 2010 (Eating and Activity Among Teens), a study examining multi-level factors associated with eating, physical activity, and weight status in adolescents in the Minneapolis/St. Paul metropolitan area of Minnesota [12], to explore the sensitivity of measures to different buffering approaches. We measured access to fast-food restaurants and convenience stores, and the percentage of park, recreational, or preserve land in 1600 m network buffers. We considered both counts and densities in examining access to restaurants and stores. Counts measure how many options one has within 1600 meters and densities the relative intensity of such options. Because the buffers vary slightly in area depending on the street patterns (e.g. they are larger in areas with a grid street pattern versus a cul-de-sac pattern), these are subtly different measures.

To locate food stores we used commercial databases (accessed through Esri Business Analyst, 2010) to locate fast-food restaurants and convenience stores. All commercial lists of businesses have inaccuracies. For example, such databases may contain businesses that are no longer operating and omit others that are. In addition, not all businesses are geocoded precisely [13,14]. However, for this study what was most important was that the same data were used to test all the buffer types. Fast-food restaurants were identified first by using North American Industrial Classification System (NAICS) codes to locate all restaurants. Then, because of problems identified earlier with miscoding of fast food in such databases [13], we identified such restaurants by searching using key words from two lists. The first list included over 60 chain restaurant names and the second list was composed of 18 key words such as “take out”, “fried”, and “pizza” [11].

We did not use fieldwork to locate food outlets because the participants were widely scattered. Triangulation against restaurant licensing is another way to check accuracy but as 51 municipalities were represented in the study and each municipality had different restaurant licensing categories and data formats such comparison would have been cumbersome. For this evaluation of buffers what mattered most was that the base data were consistent. Convenience stores, including those attached to gas stations, were located using NAICS codes. While this procedure will have missed some stores selling convenience foods (e.g., department stores), it provided a workable set of data for this comparison. Other parts of the study examined different ways of defining food stores and restaurants.

Percent park, recreation, and preserve (or “open space”) is a measure of access to recreational opportunities such as parks and was used because of its availability across municipalities. It is based on both municipal parcel data and aerial photo interpretation [15]. Data on parks alone were not consistently available for all areas where study participants resided.

Finally we examined whether the associations between these environmental measures and three outcome variables were similar or different for adolescents across different buffer types. The outcomes were self-reported frequency of fast-food purchases, fruit and vegetable consumption, and physical activity. Fast-food consumption was measured using the question: “In the past week, how often did you eat something from a fast food restaurant (like McDonald’s, Burger King, Hardee’s, etc.)?” Six response categories ranged from “never” to “more than seven times.” Fruit and vegetable consumption was measured using a semi-quantitative food frequency questionnaire specifically designed for youth [16,17]. Fruit intake (excluding juice) was estimated by summing the reported consumption of nine items; vegetable intake (excluding potatoes) was estimated by summing the reported consumption of 19 items on the questionnaire; one serving was defined as the equivalent of one-half cup. Moderate and vigorous physical activity (MVPA) was measured using questions that were adapted from the Godin Leisure-Time Exercise Questionnaire [18,19]. Adolescents were asked: “In a usual week, how many hours do you spend doing the following acti-vities: (1) strenuous exercise (e.g. biking fast, aerobics, jogging, swimming laps, soccer, rollerblading), (2) moderate exercise (e.g. walking quickly, easy bicycling, skiing, dancing, skateboarding, snowboarding)”. Response options ranged from “none” to “6+ hours a week.” The study was approved by the University of Minnesota’s Institutional Review Board for the protection of human research subjects.

## Results and discussion

### Buffer development

**Figure 1:**
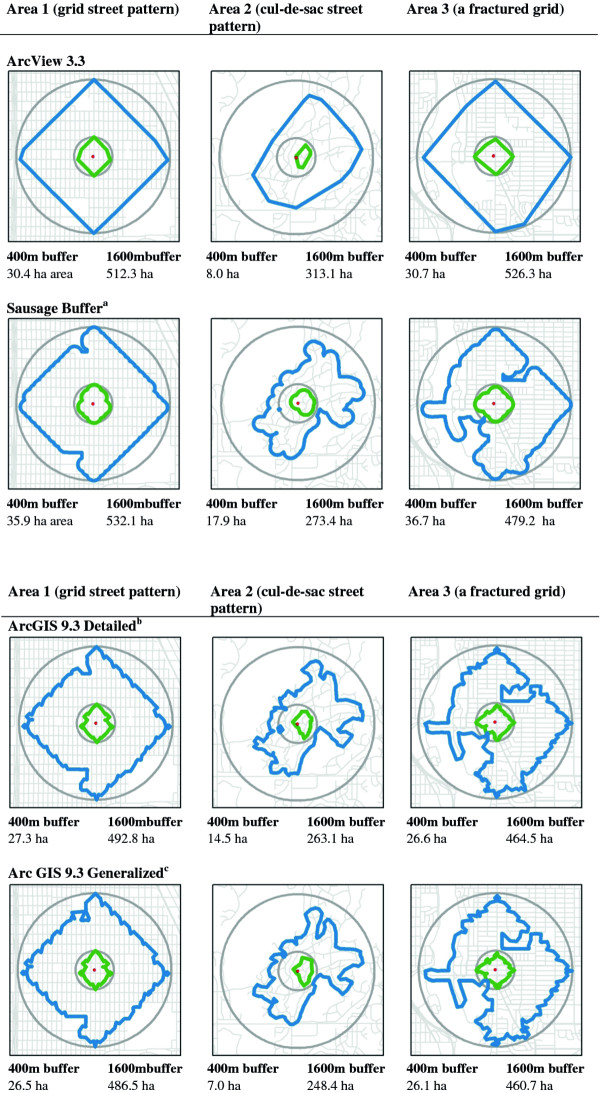
***ArcView 3.3, ArcGIS 9.3 Detailed and Generalized Buffers, and the New “Sausage”*Buffer, Compared with Circular Buffer in Different Locations at 400 m and 1600 m.**

Examples of network buffers for ArcView 3.3, ArcGIS 9.3 detailed and generalized buffers, and the sausage buffer are illustrated in Figure 1. All are drawn for street distances of 400 and 1600 meters from the central point and are compared with straight line or circular buffers of the same distance (see Figure 1).

The classic network buffers used in many studies conducted up to the mid-2000s were created using ArcView 3.3, an older program developed by Esri. This approach simply draws out distances of 1600 m on the street network and joins the dots—creating a shape that approximates a convex hull. This is a fairly simple and elegant buffer. It is important that results from any alternative can be compared with this buffer type.

The *detailed and generalized buffers* are proprietary algorithms in the ArcGIS software suite. The program has very minimal documentation on how they are created or their differences. In total, the documentation with ArcGIS version 9.3 explains: “Generalized polygons are generated quickly and are fairly accurate, except in the fringes. The generalizing of polygons may result in islands of unreached elements being covered”; “Detailed polygons model the service areas more accurately and thus may result in islands of unreached areas. Expect detailed polygons to take noticeably longer to generate than generalized polygons.”

**Figure 2:**
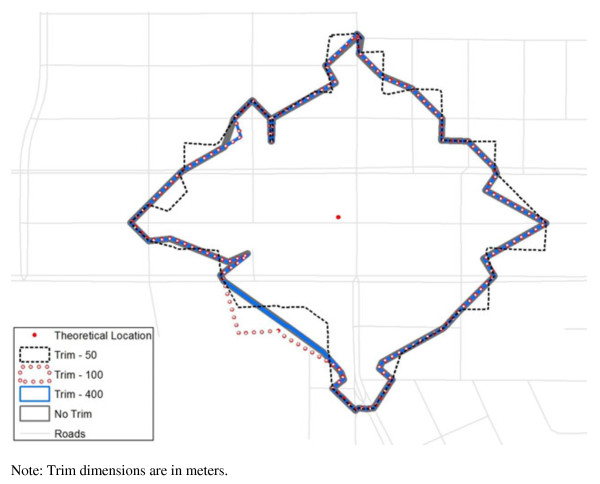
Illustration of Trim Options for Generalized Buffer at 400 m.

The only control a technician has is which kind of buffer to use (detailed, generalized), its distance, and whether to “trim” it. Further, trim is not a standard technical term and the only description, again from the program’s internal documentation, is “The polygons containing the network edges at the periphery of the service area can be further trimmed to be within the specified distance of these outer network edges. By default, this value is 100 meters.” Trim options are illustrated in terms of their shape in Figure 2—in this case ranging from no trim to 400 meters. While for this one buffer these differences are not enormous in terms of area this is an option that is not well explained in the documentation, an example of the opacity of proprietary algorithms.

As previously indicated the proposed *sausage buffer*, as illustrated, takes a starting point, extends a buffer a specified “street distance” and for a “radius” from the middle of each street. The examples in this paper use 150 meters as the radius, enough to capture nearby buildings that may be set well back from the street such as shopping centers behind a parking lot. It is possible to make this “radius” bigger or smaller. Chen et al. [20], in a study of hazards in residential areas, used 100 m. In our study of a wider variety of land uses, a larger radius was preferred [11]. With a smaller radius it is also more likely that there will be gaps or holes in the overall buffer, particularly in areas with large blocks. This happens when the buffer from the street on one side does not stretch to meet the buffers coming from the other sides leaving a donut hole in the middle of the block. However, people moving along the street network are experiencing the nearby environment it should not matter that the buffer is not measuring such block interiors. The fairly wide 150-meter radius buffer used in this paper minimizes this issue in urban areas as most blocks will be less than 300 meters across and thus the radii will overlap. In more rural locations it would mean that environments far distant from the road are not measured.

**Figure 3:**
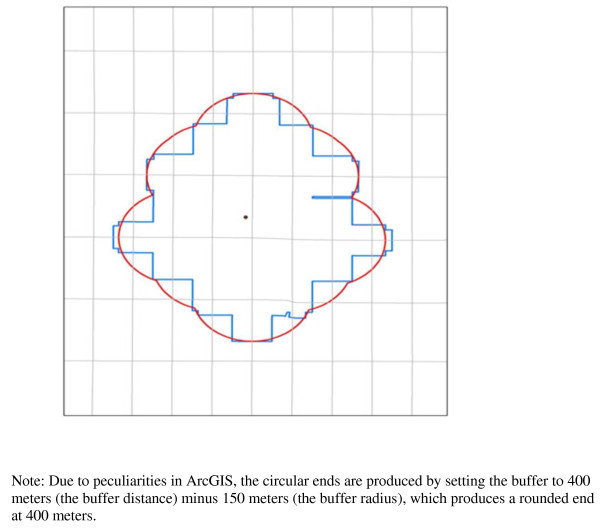
Different Options for Buffer Radius and End Points—Straight and Rounded End.

Two options for the sausage buffer’s end treatment in ArcGIS are the straight end and the rounded end (see Figure 3). We selected a rounded end as more closely approximating the distance someone can reach along a street as well as the classic buffer in ArcView 3.3. The rounded end requires an extra step so that the end of the buffer is at the required buffer distance and not rounded out beyond that. To attain the correct distance the technician sets the buffer to the buffer street distance (in this case 400 meters) minus the buffer radius (150 meters in this case), which produces a rounded end at the buffer distance (400 meters).

Overall, the ArcView 3.3 buffers in Figure 1 are the simplest in form with the fewest turns and corners, followed by the sausage buffer. In these illustrations the detailed and generalized buffers look similar. Areas of the various network buffers examined here are close in area to the sausage buffer (Figure 1).

Finally, to more fully test whether buffer sizes differ, Table 1 compares six buffer types in terms of areas, using data for the residential neighborhoods of the 2724 EAT 2010 participants. It also compares each type of buffer to the sausage buffer using ratios of mean areas. Compared with the 400 m sausage buffer, the mean areas for all other 400 m buffers are smaller (ratios of 0.7 or 0.8). In contrast, ratios of 0.9 to 1.1 were observed when comparing the sausage buffer to other buffers of 1600 and 3000 meters. That is, the sausage buffer is slightly larger than the other network buffers for the smallest size but very comparable for the larger sizes.

**Table 1:** Comparison of Areas in Hectares for Buffers at 400, 1600, and 3000 Meters

	A. Sausage Buffer	B. ArcGIS 9.3,Gen., Trim ^a, b^	Ratio of B/A	C. ArcGIS 9.3, Gen., No Trim ^a, b^	Ratio of C/A	D. ArcGIS 9.3, Detailed, Trim ^b^	Ratio of D/A	E. ArcGIS 9.3, Detailed, No Trim ^a, b^	Ratio of E/A	F. ArcView 3.3	Ratio of F/A
**400 m**^**c, d**^											
**Mean**	**33.3**	**25.6**	**0.8**	**22.4**	**0.7**	**24.8**	**0.7**	**24.5**	**0.7**	**25.5**	**0.8**
Std. dev.	4.9	4.7		6.1		5.1		5.4		6.6	
Median	35.2	26.8		24.7		26.6		26.5		28.1	
Min.	14.9	6.5		1.2		7.0		2.0		1.7	
Max.	40.3	35.8		31.1		35.1		41.0		36.0	
**1600 m**											
**Mean**	**455.0**	**408.8**	**0.9**	**416.9**	**0.9**	**400.6**	**0.9**	**416.4**	**0.9**	**469.3**	**1.0**
Std. dev.	77.4	77.6		69.7		76.8		66.7		70.0	
Median	476.9	429.8		437.0		421.2		435.1		489.8	
Min.	68.1	35.6		22.5		43.0		72.0		55.7	
Max.	577.7	531.9		525.4		530.4		533.4		589.2	
**3000 m**											
**Mean**	**1593.6**	**1495.0**	**0.9**	**1565.6**	**1.0**	**1478.0**	**0.9**	**1550.0**	**1.0**	**1758.0**	**1.1**
Std. dev.	240.0	243.7		196.1		239.8		190.4		197.7	
Median	1660.7	1557.3		1615.5		1542.2		1597.1		1806.7	
Min.	258.7	171.2		297.1		189.0		317.8		429.4	
Max.	1977.8	1871.7		1892.5		1867.3		1875.2		2166.5	

a. Gen = Generalized buffer.

b. Trim default is 100 meters—As described in the paper, the definition of trim is not given in the program’s documentation but if people use the default value it will be set at 100 m.

c. Orientation is the opposite of Table 1 and straight line buffer is not included.

d. All figures for 2724 participants for whom data were available.

### Buffer testing

Next we explored whether these varying buffer types provided different results when measuring aspects of food and physical activity environments (see Table 2). The characteristics assessed here include fast-food restaurants, convenience stores, and percent open space. Values are obviously similar, particularly for the sausage buffer and the original ArcView 3.3 network buffers. We created correlation matrices for each of the three environment measures (fast food, convenience stores, and open space)—examining the relationship of each of the six buffer types with the others. Pearson correlations among different buffer types measuring the same variables were very high—for fast food counts and densities (all greater than 0.98 and 0.99 respectively), convenience store counts and densities (all greater than 0.97), and open space area (all greater than 0.94).

**Table 2:** **Descriptive Statistics for Five Variables Measured Using Different Buffer Types for 1600 Meter Buffers**^**a**^

				Lower	Upper		
	Mean	Std	Median	Quartile	Quartile	Min	Max
**Fast Food Count**							
Sausage buffer	5.77	5.33	5	2	8	0	61
ArcView 3.3.	5.68	5.28	5	2	8	0	61
Detailed, no trim ArcGIS 9.3	5.33	5.03	5	2	7	0	60
Detailed, trimmed ArcGIS 9.3 ^b^	5.35	5.03	5	2	7	0	60
Generalized, no trim ArcGIS 9.3	5.31	5.02	5	2	7	0	60
Generalized, trimmed ArcGIS 9.3	5.37	5.03	5	2	7	0	60
**Fast Food Density count/ha**							
Sausage buffer	0.0123	0.0107	0.0105	0.0055	0.0162	0	0.1198
ArcView 3.3.	0.0118	0.0105	0.0101	0.0047	0.0158	0	0.1166
Detailed, no trim ArcGIS 9.3	0.0126	0.0115	0.0108	0.0046	0.0171	0	0.1378
Detailed, trimmed ArcGIS 9.3	0.0130	0.0117	0.0112	0.0050	0.0176	0	0.1415
Generalized, no trim ArcGIS 9.3	0.0124	0.0112	0.0107	0.0047	0.0169	0	0.1254
Generalized, trimmed ArcGIS 9.3	0.0127	0.0113	0.0109	0.0050	0.0172	0	0.1324
**Convenience Store Count**							
Sausage buffer	4.80	2.84	5	3	6	0	29
ArcView 3.3.	4.71	2.81	4	3	6	0	29
Detailed, no trim ArcGIS 9.3	4.44	2.72	4	3	6	0	27
Detailed, trimmed ArcGIS 9.3	4.46	2.71	4	3	6	0	27
Generalized, no trim ArcGIS 9.3	4.43	2.71	4	3	6	0	28
Generalized, trimmed ArcGIS 9.3	4.47	2.71	4	3	6	0	28
**Convenience Store Density**							
Sausage buffer	0.0102	0.0055	0.0099	0.0065	0.0135	0	0.0526
ArcView 3.3.	0.0098	0.0054	0.0096	0.0061	0.0127	0	0.0526
Detailed, no trim ArcGIS 9.3	0.0105	0.0060	0.0102	0.0065	0.0138	0	0.0604
Detailed, trimmed ArcGIS 9.3	0.0108	0.0059	0.0105	0.0069	0.0141	0	0.0621
Generalized, no trim ArcGIS 9.3	0.0104	0.0058	0.0102	0.0065	0.0135	0	0.0547
Generalized, trimmed ArcGIS 9.3	0.0106	0.0057	0.0104	0.0068	0.0139	0	0.0581
**Percent Open Space**							
Sausage buffer	7.4	5.5	5.6	3.9	9.6	0	55.5
ArcView 3.3.	7.8	6.5	5.7	3.8	10.0	0	69.0
Detailed, no trim ArcGIS 9.3	7.9	6.9	5.6	3.8	9.8	0	65.4
Detailed, trimmed ArcGIS 9.3	7.0	5.0	5.4	3.8	8.8	0	59.5
Generalized, no trim ArcGIS 9.3	7.5	6.4	5.4	3.6	9.5	0	56.5
Generalized, trimmed ArcGIS 9.3	6.9	4.8	5.5	3.8	8.9	0	48.3

a. Measured for N = 2724 buffers.

b. Trim is set at 100 m.

We conducted a similar analysis, not reported in a table, for the length of street covered in each kind of buffer (for the 1,600 meter buffer): the sausage buffer, ArcView 3.3 network buffer, and the ArcGIS detailed and generalized buffers (these last two measured with and without a 100 m trim). Pearson’s correlations between the sausage buffer and all other types were high (0.98-0.99). A paired comparison t-test showed that the average length of road for the sausage buffer was not significantly different to the classic ArcView 3.3. buffer. However, the detailed and generalized buffers were significantly different to the sausage buffer (p < 0.0001) having road lengths on average 7 % shorter for the generalized buffer and 11-12 % shorter for the detailed buffer.

Table 3 takes this test one step further comparing correlations between environmental measures and outcome variables, including fast-food consumption (associated with fast-food restaurant counts), fruit and vegetable consumption (associated with convenience store counts), and MVPA (associated with percent open space). Correlations were all small but positive showing a modest association between the proximity of specific types of commercial and open space areas with health behavior outcomes. For fast food and convenience stores the correlations were similar for each different type of buffer with similar levels of statistical significance of associations. There were more magnitude differences in correlations for open space though none were statistically significant and the sausage buffer produced similar results to ArcView 3.3. We also looked at densities of restaurants and stores and results were similar to those for counts. Thus, the sausage buffer finds associations where other buffers do with similar magnitudes.

**Table 3:** Correlations Between Adolescent Outcomes and Access to Fast food Restaurants, Convenience Stores, and Percent Open Space for Different Buffer Types at 1,600 Meters

Environmental measure for correlation (across)	Fast -food purchases	Fruit and vegetable consumption	Moderate and vigorous physical activity
Outcome measure for correlation (across)	Fast-food restaurant counts	Convenience storecounts	Open Space%
Buffer type (down)			
Sausage buffer	0.060*	0.045*	0.034
ArcView 3.3.	0.063*	0.042*	0.033
Arc GIS 9.3			
Detailed, no trim	0.065*	0.047*	0.034
Detailed, trimmed	0.065*	0.044*	0.025
Generalized, no trim	0.067*	0.042*	0.026
Generalized, trimmed	0.065*	0.044*	0.025
N	2715	2444	2719

* p < .05, correlation coefficient.

## Conclusions

This paper set out to answer two questions: (a) is it possible to create a buffering approach that can be replicated across software versions and computer platforms and (b) can such an approach measure the relevant aspects of the environment? The paper proposes that sausage buffers provide such an approach. Sausage buffers are a theoretically defensible way of measuring exposures akin to the experience of the street environment. Findings from various tests in the current study show that researchers can obtain results using sausage buffers that are similar to results they would obtain by using other buffering techniques. However, unlike proprietary buffering techniques, the sausage buffer approach can be replicated across software programs and versions, allowing more independence of research from specific software. While any measurement using a GIS system uses some embedded assumptions, the sausage network buffer minimizes the assumptions that need to be made. It is difficult to imagine a full-featured GIS program that could not create a buffer of a specified radius along streets a certain distance.

Developing such an approach to buffering is important for researchers. At present, corporate decisions to change buffering algorithms can alter results. Given the care researchers pay to replication in other parts of their work this is an important problem. While such changes may be modest in some cases, results may still be sensitive to changes.

The sausage buffering approach has many strengths but does not solve all problems with buffering in research related to physical activity and food environments. The most acute issue is that pedestrians and cyclists do not always move along the street network. However, many databases have incomplete information on pedestrian and bicycling paths and trails or about shortcuts through areas like parks and parking lots. This is a problem with underlying data on the movement network—not the buffering—and is an area where more resources are needed. The sausage buffer can handle buffering such paths where complete data are available.

Further research is needed to evaluate the sausage buffer as a repeatable alternative suitable for research uses with utility across programs and places. While we know that this method can be used with many software programs and it has been demonstrated in the health field using MapInfo and ArcGIS in Australia [5] and the United States (this study), others should test the buffer using with a wider range of programs. In addition, to ensure utility across places, it could be tested in different kinds of environments and with different assumptions such as radius sizes.

In order to further the field of obesity research it is important to be able to compare data across studies. Proprietary techniques can limit such comparisons if they are unique to a particular software or changed over time. The sausage buffer technique provides a solution to this problem, representing a reliable and valid alternative to proprietary algorithms used to create network buffers, and should be useful in health research. Future releases of ArcGIS could, perhaps, make the sausage buffer a more obvious option allowing researchers interested in comparative work to select it.

## Endnotes

^a^The GIS approach for creating the sausage buffers requires two steps. First, the Service Area Solver in the Network Analyst extension for ArcGIS 9.3.1 is used to calculate service areas around point features (such as study participant homes and schools). The Service Area Solver creates both polygons and lines that comprise the service area. These lines serve as the input for the second step that buffers the lines out by the buffer radius [11].

## **Abbreviations**

EAT 2010: Eating and Activity among Teens; MVPA: Moderate and vigorous physical activity; GIS: Geographic Information Systems.

## Competing interest

The authors declare that they have no competing interest.

## Authors’ contributions

AF coordinated the larger GIS protocols for the study and led in drafting the manuscript. DVR carried out the GIS measurements, developing detailed methods for creating the sausage buffers, and contributed to the manuscript. NL managed the EAT 2010 study, was involved in conceptualizing sausage buffers, and helped draft the manuscript. MW participated in the design of the study, performed the statistical analysis, and helped draft the manuscript. DNS conceived of the overall study, and participated in its design and coordination, and helped to draft the manuscript. All authors read and approved the final manuscript.
